# Risk factors of postoperative sexual function in patients with urethral diverticulum and their partners: A cohort study of 83 women

**DOI:** 10.1590/S1677-5538.IBJU.2018.0824

**Published:** 2019-12-17

**Authors:** Yi Sun, Cai Tang, Na Li, De-yi Luo, Liao Peng, Hong Shen, Qiang Wei

**Affiliations:** 1 Department of Urology, Institution of Urology, West China Hospital, Sichuan University, Guoxue, Xiang, Chengdu, China;; 2 Department of Urology, West China School of Public Health and West China Fourth Hospital, Sichuan University, Chengdu, China;; 3 Department of Pediatrics, Chengdu Tianfu New District People's Hospital, Chengdu, China

**Keywords:** Urethra, Diverticulum, Female, Male

## Abstract

**Introduction and Objective:**

Several studies have focused on the treatment and recurrence of urethral diverticulum (UD). However, few investigations have addressed sexual function in patients with UD. Therefore, we sought to examine sexual function in women affected by UD.

**Materials and Methods:**

There were 108 accepted cases involving transvaginal diverticulectomy at our institution. Ultimately, 83 women were included for further analysis, only 61 of these women had sexual partners. We collected data for the Female Sexual Function Index (FSFI) from the female patients and the Male Sexual Health Questionnaire (MSHQ) from their male partners before and after surgery.

**Results:**

Preoperatively, the UD size affected the female patient's arousal and lubrication (p=0.04), and the UD location affected their satisfaction. However, no significant between-group differences were found in the total FSFI score. For all women, sexual activity improved after surgery (p=0.0087). In addition to improvements in arousal for women with a large UD, improvements in lubrication were affected by the UD size, number and shape, increases in satisfaction scores were impacted by the UD location and shape, and pain relief was linked to the UD number and shape. Analysis of the MSHQ results revealed no between-group differences among the male partners.

**Conclusion:**

Only the UD size and location affected sexual function in women with a small UD. Surgery could improve female sexual function but did not affect the sexual function of the patient's partners.

## INTRODUCTION

Approximately 0.6-6.0% of women worldwide are impacted by urethral diverticulum (UD), and the most common symptoms of this disease include recurrent urinary tract infection (UTI), a vaginal wall mass, dysuria, dyspareunia, voiding dysfunction, and postvoid dribbling (1–3). These nonspecific symptoms present a significant diagnostic dilemma (4). Although a wide range of treatments for UD have been described, surgical excision is generally recognized as the standard therapy, with reported cure rates from 70-97% (2-6). In the literature, many postoperative complications have been reported, including UTI, superficial wound infection, stress urinary incontinence (SUI), UD recurrence, urinary fistula, and urethral stricture (7). In addition, certain reviews have reported reoperation rates as high as 10.7% (8). In our previous study, we clarified the risk factors of UD recurrence (9). We stated that many studies have researched surgical complications, but no studies have focused on the sexual function of patients with UD. Therefore, we decided to assess the influences of UD and surgical excision on sexual function in women and (if present) in their male partners.

## MATERIALS AND METHODS

After receiving institutional review board approval from the ethics committee of West China Hospital, Sichuan University, we performed a retrospective chart review of women who underwent urethral diverticulectomy at our institution. The diagnosis of UD was based on the typical triad of dysuria, dribbling, and dyspareunia. Patients presented with a wide spectrum of urinary complaints, including recurrent UTI, incontinence, pelvic pain, frequent urination, hematuria, and urinary retention. In addition, physical examinations and ultrasound imaging or magnetic resonance imaging (MRI) were performed. Women who were eventually diagnosed with carcinoma based on pathology or urethrectomy were excluded from our analysis.

From January 2009 to October 2016, there were 108 accepted cases of UD treated with transvaginal diverticulectomy. All of the patients accepted the baseline cystoscopy test to verify UD and exclude bladder masses and other diseases. Ultimately, 83 women were included for further analysis, only 61 of these women had partners, and none of those partners had sexual dysfunction, as stated by themselves. The participant's clinical characteristics were collected and recorded. Fellowship-trained urologists at our institution performed all urethral diverticulectomies in a standard manner, as previously described (9). The steps of this procedure include careful dissection, complete excision and watertight three-layer closure without tension (10). No concomitant anti-incontinence procedures were performed, and a urethral Foley catheter was routinely retained for 3-4 weeks after the surgery, in accordance with our prior description (9). When comparing changes in sexual function between patient groups, we recorded the UD-related conditions of each patient, including previous urethral surgeries (previous urethral surgery indicated recurrence), the maximum diverticulum size (diverticula smaller than 3cm were recorded as small), the number of diverticula (single or multiple), the diverticulum location(s) (proximal or mid/distal determined via MRI with the vagina divided equally into proximal and mid/distal parts), and the diverticulum shape(s), including simple or horseshoe/circumferential. A circumferential UD, such as an ostium or bulging mass extending from the bladder neck to beyond the external sphincter, affects the bladder neck. A horseshoe UD always forms a periurethral U-shape, extending around the lateral and posterior portions of the urethra, as well as anteriorly (11, 12).

All patients were instructed to return to the hospital 12 months after the surgery. The Female Sexual Function Index (FSFI) was used to assess the female patient's sexual function at baseline and at the 12-month follow-up. Additionally, the patient's male partners who agreed to participate simultaneously completed the Male Sexual Health Questionnaire (MSHQ). The FSFI is an internationally validated questionnaire that is widely used to assess sexual function (13). This questionnaire is composed of 19 questions and covers the following domains: desire, arousal, lubrication, orgasm, satisfaction, and pain. A validated Dutch version of the FSFI is available (14). The total FSFI score ranges between 2 and 36, with higher scores indicating better sexual function. The MSHQ consists of the following domains: erection, ejaculation, satisfaction, and desire. This questionnaire produces scores ranging from 0 to 5-35 (differing by domain), with higher scores indicating better sexual function (15). The MSHQ has also been validated for the Dutch population (16). The FSFI and MSHQ domain scores were calculated according to the provided guidelines.

Data were collected and imported to statistical software SPSS 18.0. First, descriptive analyses and Student's t-test were used for comparisons involving age, body mass index (BMI), parity and UD size, whereas the Mann-Whitney U test was used to assess the symptoms duration and follow-up data. Second, with respect to the analysis of outcome data, an unpaired Student's t-test was used for normally distributed data, which are presented as the mean and standard deviation. The Mann-Whitney U test was used for data with a skewed distribution, and Fisher's exact test was used for proportions. Moreover, logistic regression analysis was performed to identify factors of surgical outcomes.

## RESULTS

### Patient Characteristics

**Figure 1:**
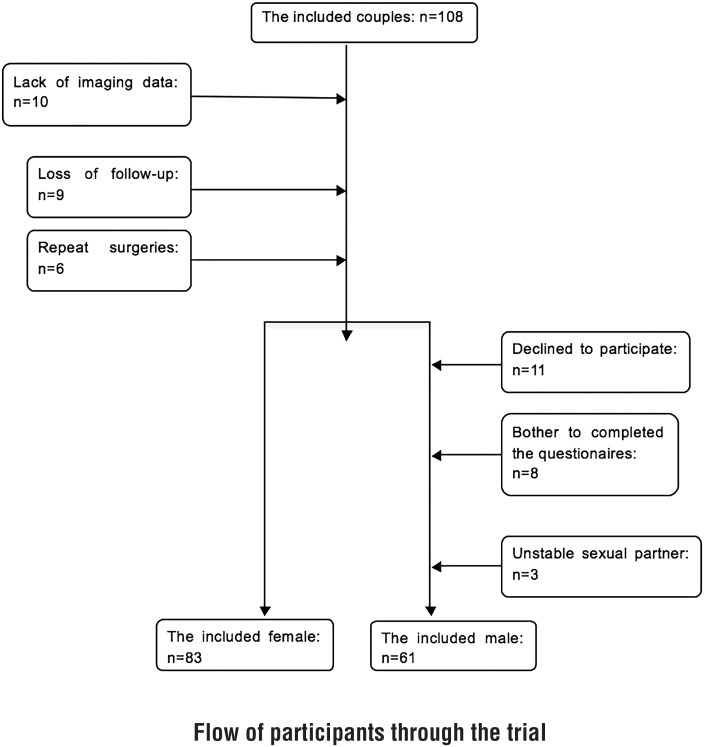
Flow of participants through the trial.

At the 12-month follow-up, 83 of the 108 female patients returned the FSFI questionnaire and were included in this study. However, only 61 of these women were accompanied by male partners who agreed to participate and returned the MSHQ. Of the 108 eligible patients, 25 were excluded for the following reasons: lack of imaging data (n=10), lost to follow-up (n=9), and repeat surgery (n=6). The patient clinical characteristics, including age at surgery, BMI, parity, symptom duration (the interval between onset and surgery), history of urethral surgery, and UD condition-related data, are presented in Table-1. The patient collection process is depicted in Figure-1.

**Table 1:** Clinical characteristics of patients.

Variables		Total (N = 83)
Age		45.1±9.1
BMI (kg/m^2^)		25.1±3.2
Parity		2.5±1.0
Duration (mean, range)		28.4 (1-120)
Previous urethral surgery		12
Size		2.79±1.3
**Location**		
	Proximal	23
	Mid	48
	Distal	12
**Shape**		
	Simple	45
	Horseshoe	21
	Circumferential	17
**Number**		
	Single	69
	Multiple	14
**Pathological results**		
	Normal urothelial tissue	12
	Chronic inflammation	36
	Acute inflammation	8
	Purulent inflammation	4
	Squamous carcinoma	8
	Atypical hyperplasia	3
	Metaplasia	3
	Absent	9

**BMI** = body mass index

### Female Sexual Function

**Figure 2:**
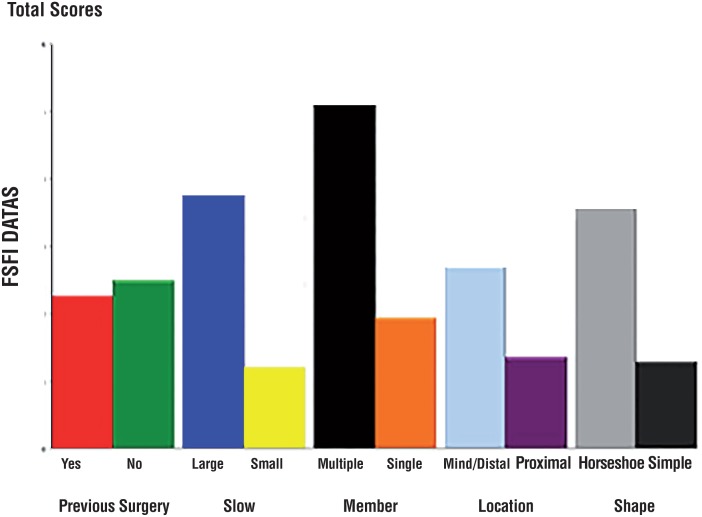
The total scores of FSFI affected by several groups.

Baseline FSFI domain scores are shown in Table-2. Patients with diverticula of different sizes reported different arousal conditions (2.13±1.01 in the large UD group and 2.61±2.12 in the small UD group, p=0.039). In addition, lubrication was affected by the UD size (2.05±2.41 in the large UD group and 2.56±2.51 in the small UD group, p=0.04). Moreover, women showed greater satisfaction when the UD was proximal to the urinary meatus than when the UD was elsewhere (2.76±2.01 in the proximal UD group and 2.04±2.0 in the mid/distal UD group, p=0.04). In particular, the total score was also affected by location (9.91±1.12 in the proximal UD group and 8.92±1.19 in the mid/distal UD group, p=0.01). Other FSFI items and the total FSFI score showed no significant differences. Changes in the FSFI score for female patients after surgery are presented in Table-3. The total FSFI score for all women markedly improved after surgery (p=0.0087). This finding indicates that surgery could help revive patient's sexual function rather than merely treat urinary symptoms. With respect to items examined for different groups of patients, arousal, lubrication, satisfaction and pain improved by varying degrees. First, the increase in the arousal score was significantly greater for patients with a large UD than for patients with a small UD (0.61±1.42 versus 0.15±1.81, p=0.042). Second, the increase in the lubrication score was greater in the large UD group than in the small UD group (0.84±2.07 versus 0.12±1.98, p=0.031); this increase was larger in the multiple UD group than in the single UD group (0.80±0.42 versus 0.51±0.22, p=0.015) and in the horseshoe/circumferential UD group than in the simple UD group (0.90±0.26 versus 0.59±0.27, p=0.008). In addition, the increase in the satisfaction score was better in the mid/distal UD group than in the proximal UD group (0.70±0.57 versus 0.25±0.63, p=0.017) and in the horseshoe/circumferential UD group than in the simple UD group (1.01±0.44 versus 0.10±0.21, p=0.003). Moreover, the decrease in the pain score was greater in the multiple UD group than in the single UD group (-0.78±2.2 versus-0.25±1.88, p=0.002) and in the horseshoe/circumferential UD group than in the simple UD group (-0.81±0.75 versus −0.27±0.55, p=0.012). The total score exhibited greater improvement in the large UD group than in the small UD group (3.47±1.35 versus 2.20±0.78 p <0.005), in the multiple UD group than in the single UD group (3.67±1.82 versus 2.66±1.10 p=0.03), and in the horseshoe/circumferential UD group than in simple UD group (p=3.30±1.20 versus 2.42±1.91 p=0.008). Changes in the total FSFI score are shown in Figure-2.

**Table 2:** The change of FSFI data after operation.

		Desire	Arousal	Lubrication	Orgasm	Satisfaction	Pain	Total
Previous urethral surgery								
	Yes (n=12)	0.20±0.28	0.15±0.23	0.55±0.27	0.47±0.15	0.40±0.61	-0.87±0.27	2.63±0.99
	No (n=71)	0.15±0.31	0.41±0.41	0.64±0.27	0.38±0.28	0.55±0.58	-0.71±0.68	2.85±1.30
	p	0.73	0.13	0.44	0.45	0.56	0.59	0.70
**Size**								
	Large (n=41)	0.24±0.37	0.45±0.48	0.83±0.22	0.37±0.30	0.66±0.63	-0.93±0.73	3.47±1.35
	Small (n=42)	0.08±0.21	0.32±0.28	0.44±0.15	0.40±0.24	0.41±0.50	-0.54±0.48	2.20±0.78
	p	0.06	0.23	<0.005	0.70	0.12	0.02	<0.005
**Number**								
	Multiple (n=14)	0.20±0.30	0.40±0.47	0.80±0.42	0.31±0.27	0.49±0.66	-1.47±0.94	3.67±1.82
	Single (n=69)	0.15±0.32	0.38±0.39	0.60±0.22	0.40±0.27	0.54±0.57	-0.59±0.48	2.66±1.10
	p	0.66	0.90	0.04	0.37	0.81	<0.005	0.03
**Location**								
	Mid/Distal (n=60)	0.18±0.33	0.42±0.43	0.67±0.25	0.55±0.45	0.50±0.57	-0.69±0.63	2.82±1.20
	Proximal (n=23)	0.05±0.18	0.22±0.19	0.46±0.28	0.35±0.20	0.65±0.63	-0.91±0.74	2.85±1.63
	p	0.22	0.13	0.02	0.03	0.45	0.31	0.95
**Shape (n=38/45)**								
	Horseshoe/circumferential	0.09±0.22	0.40±0.38	0.68±0.26	0.40±0.31	1.05±0.44	-0.68±0.75	3.30±1.20
	Simple	0.21±0.36	0.37±0.42	0.59±0.27	0.37±0.23	0.10±0.21	-0.77±0.55	2.42±1.19
	p	0.15	0.74	0.21	0.72	<0.005	0.58	0.008

**Table 3:** Mean change in MSHQ domain scores before and after surgery.

		Erection	Erection bother	Ejaculation	Ejaculation bother	Satisfaction
Previous urethral surgery						
	Yes (n=12)	1.80±0.40	-1.00±0.89	7.00±2.45	-1.00±0.63	2.60±0.49
	No (n=71)	2.22±0.18	-0.84±0.77	3.31±2.14	-1.20±0.74	1.45±1.04
	p	0.43	0.66	<0.005	0.56	0.02
**Size**						
	Large (n=41)	1.96±0.90	-1.08±0.78	4.27±2.44	-1.19±0.88	1.92±0.87
	Small (n=42)	2.39±1.29	-0.64±0.73	3.07±2.37	-1.18±0.55	1.21±1.04
	p	0.16	0.04	0.07	0.95	0.01
**Number**						
	Multiple (n=14)	1.67±0.47	-1.11±0.87	6.22±2.20	-1.22±0.63	2.33±0.47
	Single (n=69)	2.29±1.20	-0.80±0.76	3.67±2.39	-1.18±0.75	1.55±1.04
	p	0.13	0.28	<0.005	0.87	0.01
**Location**						
	Mid/Distal (n=60)	2.29±1.08	-0.90±0.78	3.62±2.54	-1.19±0.76	1.64±1.07
	Proximal (n=23)	1.83±1.27	-0.67±0.78	3.75±1.96	-1.17±0.58	1.25±0.97
	P	0.23	0.36	0.87	0.92	0.26
**Shape (n=38/45)**						
	Horseshoe/circumferential	0.28±1.74	-0.02±0.57	4.27±2.44	-0.17±0.41	1.92±0.87
	Simple	0.22±2.07	-0.02±0.4	3.07±2.26	-0.17±0.26	1.21±1.10
	p	0.51	0.3	0.07	1.93	0.01

**MSHQ**= Male Sexual Health Questionnaire

### Male Sexual Function

As shown in Table-4, the males examined in this study had similar MSHQ domain scores at baseline, and the UD conditions of the female partners did not influence their male partner's MSHQ domain scores. Changes in the MSHQ domain scores after surgery are presented in Table-5. At 12 months, only 43 men completed and returned the MSHQ, none of the males included in this study reported having premature ejaculation, erectile dysfunction or any other sexual dysfunction. Three women did not have a stable sexual partner, 21 men found that completing the MSHQ was too troublesome, and three men declined to participate in the analysis (Figure-1). Our results based on data collected at the 12-month follow-up demonstrate no differences among the couples. Thus, surgery in females for UD might have no effect on their male partners.

**Table 4:** The FSFI data in the pre-operative period

		Desire	Arousal	Lubrication	Orgasm	Satisfaction	Pain	Total
Previous urethral surgery								
	Yes (n=12)	2.30±0.24	2.55±0.49	2.60±0.45	2.53±0.70	2.67±0.41	2.60±0.66	10.05±0.89
	No (n=71)	2.59±0.56	2.52±0.33	2.34±0.83	2.41±0.48	2.25±0.57	2.44±0.44	9.67±1.22
	p	0.22	0.82	0.46	0.19	0.09	0.44	0.47
**Size**								
	Large (n=41)	2.53±0.56	2.55±0.36	2.55±0.72	2.37±0.53	2.44±0.59	2.57±0.49	9.87±1.10
	Small (n=42)	2.59±0.52	2.49±0.33	2.21±0.83	2.48±0.46	2.16±0.52	2.36±0.40	9.57±1.25
	P	0.68	0.53	0.10	0.43	0.06	0.08	0.33
**Number**								
	Multiple (n=14)	2.33±0.52	2.5±0.28	2.23±1.14	2.44±0.48	2.04±0.64	2.36±0.44	9.2±1.70
	Single (n=69)	2.6±0.53	2.52±0.35	2.39±0.72	2.42±0.50	2.34±0.54	2.48±0.46	9.8±1.05
	P	0.17	0.85	0.58	0.91	0.16	0.46	0.17
**Location**								
	Mid/Distal (n=60)	2.40±0.63	2.45±0.31	2.00±1.00	2.37±0.58	2.03±0.55	2.33±0.54	8.92±1.19
	Proximal (n=23)	2.60±0.52	2.54±0.36	2.46±0.73	2.44±0.48	2.36±0.56	2.49±0.44	9.91±1.12
	P	0.25	0.44	0.07	0.65	0.08	0.29	0.01
**Shape (n=38/45)**								
	Horseshoe/circumferential	2.64±0.42	2.56±0.37	2.34±0.72	2.43±0.47	2.28±0.62	2.55±0.42	9.71±1.03
	Simple	2.49±0.62	2.49±0.33	2.39±0.88	2.42±0.53	2.30±0.54	2.39±0.48	9.71±1.33
	p	0.28	0.48	0.83	0.97	0.89	0.18	0.99

**FSFI** = Female Sexual Function Index

**Table 5:** The MSHQ domain scores in the pre-operative period

		Erection	Erection bother	Ejaculation	Ejaculation bother	Satisfaction
**Previous urethral surgery**						
	Yes (n=12)	7.80±0.40	4.40±0.49	19.0±1.79	3.60±0.80	14.00±2.45
	No (n=71)	7.31±1.77	4.29±0.71	18.84±4.86	3.78±0.69	11.67±3.00
	p	0.54	0.73	0.94	0.60	0.10
**Size**						
	Large (n=41)	6.84±2.11	4.08±0.69	17.65±5.50	3.96±0.72	12.04±4.02
	Small (n=42)	7.82±1.02	4.5±0.64	19.96±3.45	3.57±0.63	11.75±1.73
	P	0.03	0.02	0.07	0.04	0.73
**Number**						
	Multiple (n=14)	7.78±0.42	4.56±0.53	19.22±1.64	3.89±0.78	13.33±2.34
	Single (n=69)	7.27±1.84	4.24±0.71	18.78±5.06	3.73±0.69	11.60±3.11
	P	0.41	0.22	0.80	0.55	0.12
**Location**						
	Mid/Distal (n=60)	7.19±1.75	4.29±0.66	18.12±4.53	3.79±0.71	11.81±3.35
	Proximal (n=23)	7.92±1.31	4.33±0.78	21.42±4.12	3.67±0.65	12.17±1.11
	p	0.19	0.84	0.03	0.61	0.72
**Shape (n=38/45)**						
	Horseshoe/circumferential	6.85±2.12	4.08±0.69	17.65±5.50	3.96±0.72	12.04±4.02
	Simple	7.82±1.02	4.50±0.64	19.96±3.45	3.57±0.63	11.75±1.73
	p	0.03	0.02	0.07	0.04	0.73

**MSHQ** = Male Sexual Health Questionnaire

## DISCUSSION

Although urethral diverticulectomy is a rare procedure, it is reportedly being performed more frequently (7). Patients are usually diagnosed with UD based on typical symptoms, including a vaginal wall mass, dysuria, postvoid dribbling, and dyspareunia, although this condition has a wide range of presentations (1). According to some studies, UD is a focal epithelium-lined outpouching that occurs at the urethra, this feature is recognized by researchers as originating from a dilated periurethral gland (17). Female patients with UD are frequently diagnosed long after they have become symptomatic, this phenomenon may be attributed to the varying presentations of UD, which may delay definitive treatment (4, 18). There are several treatment options for UD, including surgery, marsupialization, partial ablation, or urethral diverticulectomy. Among these options, diverticulectomy is considered the gold standard (19). Various types of conservative management options, such as endoscopic or open incision, drainage and coagulation, have been described in previous studies (20–22). However, these treatments have not been widely adopted due to the high recurrence rate, the risk of persistent infection and missed malignancy. With respect to the key aspect of these operations, all procedures should fully excise the UD, including the ostium, and the repair should be performed with multiple (typically three) overlying tissue layers (23). Several studies have addressed the efficacy and safety of transvaginal diverticulectomy, but studies have rarely focused on sexual function in females surgically treated for UD. In this study, we included 83 women who underwent transvaginal diverticulectomy at our medical center, and we collected and recorded FSFI and MSHQ scores before and after the surgery.

According to certain reports, pelvic floor disorders are strongly related to sexual dysfunction, although other factors, such as advanced age, postmenopausal status, and body image, may be important confounders (24, 25). However, few studies have investigated the effect of urethral di- verticulectomy on this condition.

The study results indicate that UD has no remarkable impact on female patient's sexual function or the sexual function of the patient's male partners. There were no significant differences in the FSFI domain scores among females with UD. However, small effects were observed in individual domains, such as arousal, lubrication and satisfaction. Certain factors, such as the UD size, could affect arousal and lubrication. In the interviews that we conducted, participants said that when they engaged in foreplay, the mass located at the urethra caused a pricking sensation, and urine overflowed from the diverticulum. In addition, females with a proximal UD could feel increased satisfaction. We considered that a proximal UD may be able to affect clitoral stimulation and causes increased satisfaction, however, this phenomenon still requires further exploration. In addition, we found that the sexual function of the patient's male partners did not significantly differ, regardless of the patient's total and individual domain scores. According to our interviews, the male partners frequently overlooked aspects of the female partner's UD condition. The diagnosis of UD is considered difficult, as certain studies have reported, due to the array of nonspecific symptoms associated with this condition (26). Moreover, the UD size, number, and shape could affect sexual function after surgery. Thus, additional research should be performed to investigate the mechanism underlying the effects of these factors on UD. However, the efficacy of transvaginal diverticulectomy was confirmed by the results of our study. Our results can be compared with those of previous studies addressing improvements in urinary symptoms following surgery for UD. There may be other factors that can impact female sexual function, such as the vaginal level of estrogen or other hormones. In this study, we tested the blood estrogen level of patients older than 45 and found no significant differences. Santoro et al. reported that the vaginal estrogen level could improve female sexual function (27), but we did not test the vaginal estrogen level in this study, however, in future work, we will focus on this point.

Several limitations of this study should be addressed. First, the study design was a retrospective case series. Therefore, the results are restricted by the inherent characteristics of a retrospective design. Future investigations should consist of randomized controlled studies. Moreover, the sample size in our study was small due to the low morbidity rate of UD. In addition, disease recurrence can occur for various reasons, and confirming the effect of recurrence on female sexual function is necessary. However, in our study, only eight subjects required reoperation. According to our previous study, women with multiple and proximal diverticula could be at an increased risk of recurrence (9). In our study, these two factors were examined after surgery. The results indicate that the number of diverticula could affect female patient's FSFI scores, particularly in the two domains of lubrication and pain. The UD location could also impact female patient's sexual satisfaction. These two items should receive the most research attention.

## CONCLUSIONS

Preoperatively, the UD size can be associated with differences in arousal and lubrication. Moreover, the UD location can affect female patient's sexual satisfaction. According to the total FSFI score, female sexual activity improved after surgery. The sexual activity of females could be affected by the UD size, number, shape, and location. However, none of the examined factors influenced the sexual function of the patients’ male partners. Comparison of data among the partners of female patients in different groups revealed no differences impacting the MSHQ domain scores.

## ABBREVIATIONS

UD: Urethral diverticulum
UTI: Urinary tract infection
SUI: Stress urinary incontinence
FSFI: Female Sexual Function Index
MSHQ: Male Sexual Health Questionnaire
